# Forkhead box transcription factor FOXO3a suppresses estrogen-dependent breast cancer cell proliferation and tumorigenesis

**DOI:** 10.1186/bcr1872

**Published:** 2008-02-29

**Authors:** Yiyu Zou, Wen-Bin Tsai, Chien-Jui Cheng, Chiun Hsu, Young Min Chung, Pao-Chen Li, Sue-Hwa Lin, Mickey CT Hu

**Affiliations:** 1 Department of Medicine, Albert Einstein College of Medicine, East 210th Street, Bronx, New York, New York 10467, USA; 2 Molecular and Cellular Oncology, University of Texas MD Anderson Cancer Center, Holcombe Blvd, Houston, Texas 77030, USA; 3 Department of Pathology, Taipei Medical University and Hospital, Wu Shin Street, Taipei, 11049, Taiwan; 4 Department of Oncology, National Taiwan University Hospital, Chung-Shan South Road, Taipei, 100, Taiwan; 5 Molecular Pathology, University of Texas MD Anderson Cancer Center, Holcombe Blvd, Houston, Texas 77030, USA

## Abstract

**Introduction:**

Estrogen receptors (ERs) play key roles in breast cancer development and influence treatment outcome in breast cancer patients. Identification of molecules that regulate ER function may facilitate development of breast cancer treatment strategies. The forkhead box class O (FOXO) transcription factor FOXO3a has been suggested to function as a tumor suppressor in breast cancer. Using protein-protein interaction screening, we found that FOXO3a interacted with ER-α and ER-β proteins in the human breast carcinoma cell line MCF-7, suggesting that there exists a crosstalk between the FOXO3a and ER signaling pathways in estrogen-dependent breast cancer cells.

**Methods:**

The interaction between FOXO3a and ER was investigated by using co-immunoprecipitation and immunoblotting assays. Inhibition of ER-α and ER-β transactivation activity by FOXO was determined by luciferase reporter assays. Cell proliferation in culture was evaluated by counting cell numbers. Tumorigenesis was assessed in athymic mice that were injected with MCF-7 cell lines over-expressing FOXO3a. Protein expression levels of cyclin-dependent kinase inhibitors, cyclins, ERs, FOXM1, and the proteins encoded by ER-regulated genes in MCF-7 cell lines and breast tumors were examined by immunoblotting analysis and immunohistochemical staining.

**Results:**

We found that FOXO3a interacted with ER-α and ER-β proteins and inhibited 17β-estradiol (E2)-dependent, ER-regulated transcriptional activities. Consistent with these observations, expression of FOXO3a in the ER-positive MCF-7 cells decreased the expression of several ER-regulated genes, some of which play important roles in cell proliferation. Moreover, we found that FOXO3a upregulated the expression of the cyclin-dependent kinase inhibitors p21Cip1, p27Kip1, and p57Kip2. These findings suggest that FOXO3a induces cell growth arrest to effect tumor suppression. FOXO3a repressed the growth and survival of MCF-7 cells in cell culture. In an orthotopic breast cancer xenograft model in athymic mice, over-expression of FOXO3a in MCF-7 cells suppressed their E2-induced tumorigenesis, whereas knockdown of FOXO3a in MCF-7 resulted in the E2-independent growth.

**Conclusion:**

Functional interaction between FOXO3a and ER plays a critical role in suppressing estrogen-dependent breast cancer cell growth and tumorigenesis *in vivo*. This suggests that agents that activate FOXO3a may be novel therapeutic agents that can inhibit and prevent tumor proliferation and development in breast cancer.

## Introduction

Breast cancer is the most common malignancy diagnosed among women worldwide, and it is the second leading cause of cancer death [1]. Approximately 70% of human breast cancers express estrogen receptors (ERs) [2-4]. Many ER-α-positive human breast cancer cells require estrogen for proliferation and undergo apoptotic cell death when they are deprived of it [5]. Clinically, the presence of ER-α in breast cancer is viewed as a good prognostic factor, being associated with a lower risk for relapse and better overall disease-free survival [6]. Indeed, ER-α is a major target for endocrine therapy [7], and functional ER-α protein is both sufficient and necessary to predict responsiveness to such therapy in a high proportion of breast tumors. Thus, assessment of ER status has become standard practice in the clinical management of breast cancer [8,9], with hormonal intervention offered to patients with ER-α-positive tumors.

Current endocrine therapies for ER-α-positive breast cancer target the action of estrogen on breast cancer cells by using selective ER modulators such as tamoxifen [7,10], aromatase inhibitors such as exemestane [11], or pure antiestrogens such as fulvestrant [12]. However, only about 50% of ER-positive tumors respond to currently available hormonal therapies, and most tumors that initially respond eventually become resistant to endocrine therapy, even though ER may still be present in the tumor tissue [13]. Attempts to prevent or reverse antiestrogen resistance have been hampered by the lack of knowledge of the signaling mechanisms that underlie the regulation of ER function.

The cellular and molecular events that regulate ER-α and ER-β protein expression and function are poorly understood. Expression of ER-α may not be regulated genetically; for example, lack of expression of ER-α generally is not associated with physical loss of the ER-α gene [14]. However, ER-α expression can be regulated through epigenetic modification, for instance methylation at the promoter [15], by post-translational modifications, or through direct interaction with corepressor proteins that repress ER-α-mediated transcriptional activity [16,17]. Less is known of the regulation of expression and function of ER-β in breast cancer cells and tissues. Additional information on the cellular and molecular events that regulate ER-α and ER-β protein expression and function is needed.

FOXO3a, which is one of the forkhead box class O (FOXO) transcription factors, is a key tumor suppressor in breast cancer [18]. The function of FOXO3a is regulated mainly by nuclear translocation. In general, FOXO factors in animal cells are regulated by Akt or other kinases, which phosphorylate them at conserved serine/threonine residues [18-20]. This phosphorylation leads to the release of the FOXO transcription factors from the DNA and translocation of those factors to the cytoplasm, where 14-3-3 protein binds to the phosphorylated FOXO factors and retains them as inactive proteins in the cytoplasm. However, in the absence of stimulation from survival signals, Akt is inactivated in quiescent cells, which results in retention of FOXO factors in the nucleus. In addition to Akt, IκB kinase (IKK)-β is also important in regulating FOXO3a localization [18,21].

Nuclear FOXO has been shown to upregulate the expression of specific target genes that modulate the cell metabolic state or oxidative stress or aging [18-23], those that control cell cycle progression such as cyclin-dependent kinase (CDK) inhibitors [18,24-26], or those that regulate the mitotic program such as cyclin B and Polo-like kinase [18,26]. Downregulation of cyclin D has been implicated in FOXO-induced cell cycle inhibition in some cancer cell lines [27]. Nuclear FOXO has also been reported to induce cellular apoptosis through upregulation of Fas ligand (Fas-L) [28], Bim [29-31], and tumor necrosis factor-related apoptosis inducing ligand [32,33]. Changes in FOXO function could tip the balance between cellular differentiation and neoplastic transformation [18-20].

Although FOXO3a reportedly can suppress cell growth and tumorigenesis in ER-negative breast cancer cells [18,21], whether FOXO3a also regulates cell proliferation or tumorigenesis in ER-positive breast tumors is unknown. Here we report our discovery that FOXO3a interacts with ERs. We found that FOXO3a interacts with both ER-α and ER-β and inhibits their transcriptional activities. Gene expression profiling with a DNA microarray suggested that FOXO3a inhibits the expression of ER target genes. FOXO3a suppressed proliferation of MCF-7 breast cancer cells by inducing the expression of key CDK inhibitors and reducing the expression of cyclin D_1_. Moreover, FOXO3a suppressed tumorigenesis of E2-induced tumorigenesis of MCF-7 cells in an animal orthotopic breast tumor model, suggesting that FOXO3a plays a critical tumor-suppression role in estrogen-dependent breast cancer.

## Materials and methods

### Cell culture and cell lines

Cells were cultured in Dulbecco's modified Eagle's medium/F12 medium supplemented with 10% or 5% fetal bovine serum (FBS; Atlanta Biologicals, Lawrenceville, GA, USA) or phenol red-free Dulbecco's modified Eagle's medium/F12 medium supplemented with 5% charcoal-stripped fetal bovine serum (Atlanta Biologicals, Lawrenceville, GA), with or without 100 nmol/l 17β-estradiol (E2; Sigma-Aldrich, St. Louis, MO, USA). A MCF-7 and 293T cells were obtained from the American Type Culture Collection (Rockville, MA, USA). MCF-7 stable cell lines over-expressing FOXO3a (MCF7-FO) were generated by retroviral transduction with the pBabe vector containing hemagglutinin (HA)-tagged FOXO3a cDNA under the transcriptional control of the murine leukemia virus long terminal repeat [34] and a puromycin-resistant gene. This pBabe-HA-FOXO3a construct was created by ligating a *BamH*I-*Xho*I fragment of HA-FOXO3a into a pBabe-puro vector cut with *BamH*I and *Sal*I. MCF-7 control (MCF7-C) cell lines were established with retroviruses containing the pBabe vector without a cDNA insert. After puromycin selection (0.2 to 1.0 μg/ml), MCF7-FO pooled clones (MCF7-FO) and individual clones (FO10, FO33, and FO41), vector control pooled clones (MCF7-C,) and individual clones (C4, C5, and C12) were selected. MCF-7 stable cell line with FOXO3a downregulated (MCF7-d8_pa) was generated by retroviral transduction with a retroviral construct containing small-hairpin RNA interference targeting FOXO3a (V2HS_169297; Open Biosystems, Huntsville, AL, USA). After puromycin selection (0.2 to 1.0 μg/ml), MCF7-knockdown pooled clones (MCF7-d8_pa) were selected.

### Antibodies

Antibodies against FOXO3a (FKHRL1, sc-11351), cyclin D_1_, complement C3, and β-tubulin were purchased from Santa Cruz Biotechnology (Santa Cruz, CA, USA). Antibodies against cathepsin D, progesterone receptor (PgR), and pS2 were purchased from Novocastra Laboratories (Newcastle Upon Tyne, UK). Antibodies against p27Kip1, p21Cip1, p57Kip2, and cyclin E were obtained from BD Biosciences (San Jose, CA, USA). Antibodies against ER-α and ER-β were purchased from Upstate USA (Charlottesville, VA, USA). Finally, antibodies against HA and β-actin were purchased from Roche Applied Science (Indianapolis, IN, USA) and Sigma (St. Louis, MO, USA), respectively.

### Antibody array and DNA microarray

Protein-protein interactions were screened using an AntibodyArray from Hypromatrix, Inc. (Worcester, MA, USA). The array was incubated with whole-cell lysates of MCF7-FO33 cells, followed by a horseradish peroxidase-conjugated anti-HA monoclonal antibody, in accordance with the manufacturer's instructions. Proteins were then visualized using an enhanced chemiluminescence visualization kit from Santa Cruz Biotechnology. Of several positive antibody candidates on the membrane of the AntibodyArray, we identified a positive signal at the spot that was immobilized with an antibody against ER-α.

### Immunoprecipitation and immunoblotting

These experiments were conducted as described previously [21] with some modifications. Briefly, cells were washed twice with phosphate-buffered saline (PBS) and lysed with lysis buffer at 4°C for 20 minutes. After being sonicated with an ultrasound sonicator, whole lysates were centrifuged at 16,000 *g *for 10 minutes to remove cell debris. Total protein concentration was determined using a Bio-Rad kit (Hercules, CA, USA) using bovine serum albumin as a standard. Samples were first precleared with a nonspecific IgG control. Precleared lysates were then incubated with a specific antibody and rotated at 4°C overnight followed by the addition of 25 μl of 50% protein A- or protein G-sepharose slurry with rotation for 1 hour. Protein A or protein G beads were collected and washed with lysis buffer four times. Immunoprecipitation samples were resolved by SDS-PAGE and analyzed by immunoblotting. The protein samples were subjected to SDS-PAGE and transferred onto nitrocellulose or polyvinylidene difluoride membranes. The membranes were blocked with 5% nonfat dry milk or bovine serum albumin in PBS containing 0.05% Tween 20 and incubated with primary antibodies and then with horseradish peroxidase-conjugated secondary antibodies, in accordance with the manufacturer's instructions. The immunoblots were visualized using an enhanced chemiluminescence visualization kit (Santa Cruz Biotechnology).

### DNA transfection and cell proliferation assay

Transient DNA transfection of MCF-7 or 293T cells was performed as described previously [21,31] with the DNAs or with an optimal ratio of DNA mixtures containing 0.1 μg ER-responsive element (ERE)-luc (luciferase reporter), 0.3 μg ER-α or ER-β, 0.9 μg HA-FOXO3a or Flag-FOXO1a, 5.4 μg IKK-β [21] or control vector, and 0.01 μg pRL-TK (Promega, Madison, WI, USA), plus Lipofectamine 2000 (Invitrogen, Carlsbad, CA, USA). Cell proliferation was measured by trypan blue staining and direct cell counting.

### Tumorigenesis and growth of breast tumors *in vivo*

To determine tumorigenicity and establish orthotopic breast cancer animal models, female athymic (*nu*/*nu*) nude mice were purchased from the NCI Frederick Cancer Research Center (Frederick, MD, USA) and maintained aseptically in an athymic animal room. A 0.72 mg E2 60-day release pellet (Innovative Research of America, Sarasota, FL, USA) was implanted subcutaneously on the dorsal side of each mouse 1 day before tumor cell implantation to support the growth of the estrogen-dependent MCF-7 cell derived tumors [35]. For tumor cell implantation, MCF7-FO or control (MCF7-C) cells in log-phase growth were harvested, washed with PBS, and resuspended in PBS. Then 2 × 10^6 ^cells in 0.25 ml of the mixture were injected into the mammary fat pads of female athymic mice, as described previously [21,35]. Tumors were examined twice weekly; length, width, and thickness measurements were obtained with calipers and tumor volumes were calculated. Data are presented as means and standard deviations of three experiments with 10 mice in each group. All procedures were performed in compliance with the guidelines of the University of Texas MD Anderson Cancer Center Institutional Animal Care and Use Committee and the US National Institutes of Health.

### Immunohistochemical staining

Immunohistochemical staining was performed as described previously [21]. Breast tumors were excised from MCF-7 tumor bearing mice 35 days after inoculation of the test or control cells. Five independent tumors (each from a different mouse) were taken from MCF7-FO33 and MCF7-C5 groups for testing. Tumor samples were fixed in formalin, sectioned, placed on slides, and incubated with specific antibodies. Sections were then treated with biotin-conjugated secondary antibody followed by avidin biotin-peroxidase complex and amino-ethyl carbazole as a chromogen.

### Statistical analysis

Data are expressed as means ± standard deviations from at least three determinations. The statistical significance of differences in cell proliferation and tumor growth between two groups was analyzed with two-sided unpaired Student's *t-*tests when the variances were equal, or with Welch's corrected *t-*tests when the variances were unequal, using Graphpad statistical software (San Diego, CA, USA). All statistical tests were two-sided, and *P *values less than 0.05 were considered statistically significant.

## Results

### Identification of FOXO3a as an ER-α-interacting protein

**Figure 1:**
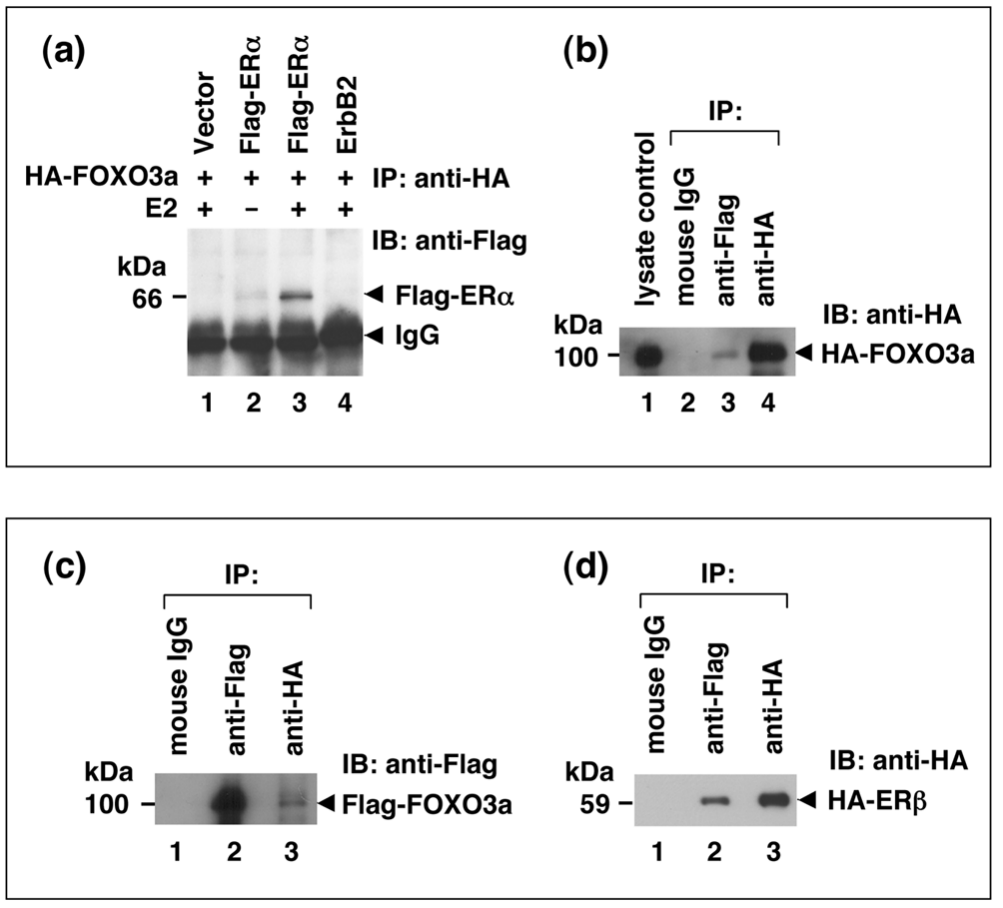
FOXO3a interacts with ER-α and ER-β. **(a) **Total lysates of 293T cells co-transfected with hemagglutinin (HA)-tagged forkhead box class O (FOXO)3a plus Flag-estrogen receptor (ER)-α (lanes 2, 3) or an empty vector (lane 1) or an ErbB2 expression vector (lane 4), in the presence or absence of 17β-estradiol (E2), were analyzed by immunoprecipitation (IP) with an anti-HA antibody followed by immunoblotting (IB) with an anti-Flag tag antibody. **(b) **The same lysates of 293T cells cotransfected with HA-FOXO3a and Flag-ER-α were subjected to reciprocal IP with an anti-Flag (lane 3) or control IgG (IgG; lane 2) or anti-HA (positive control, lane 4) followed by IB with an anti-HA. **(c) **FOXO3a associates with ER-β. Total lysates of 293T cells co-transfected with the Flag-FOXO3a and HA-ER-β expression vectors in the presence of E2 were analyzed by the same IP/IB analysis (IP: control IgG or anti-Flag [positive control] or anti-HA [lane 3]; IB: anti-Flag). **(d) **The same lysates of 293T cells co-transfected with the Flag-FOXO3a and HA-ER-β expression vectors were subjected to reciprocal IP with a control IgG or anti-Flag (lane 2) or anti-HA (positive control) followed by IB with an anti-HA.

**Figure 2:**
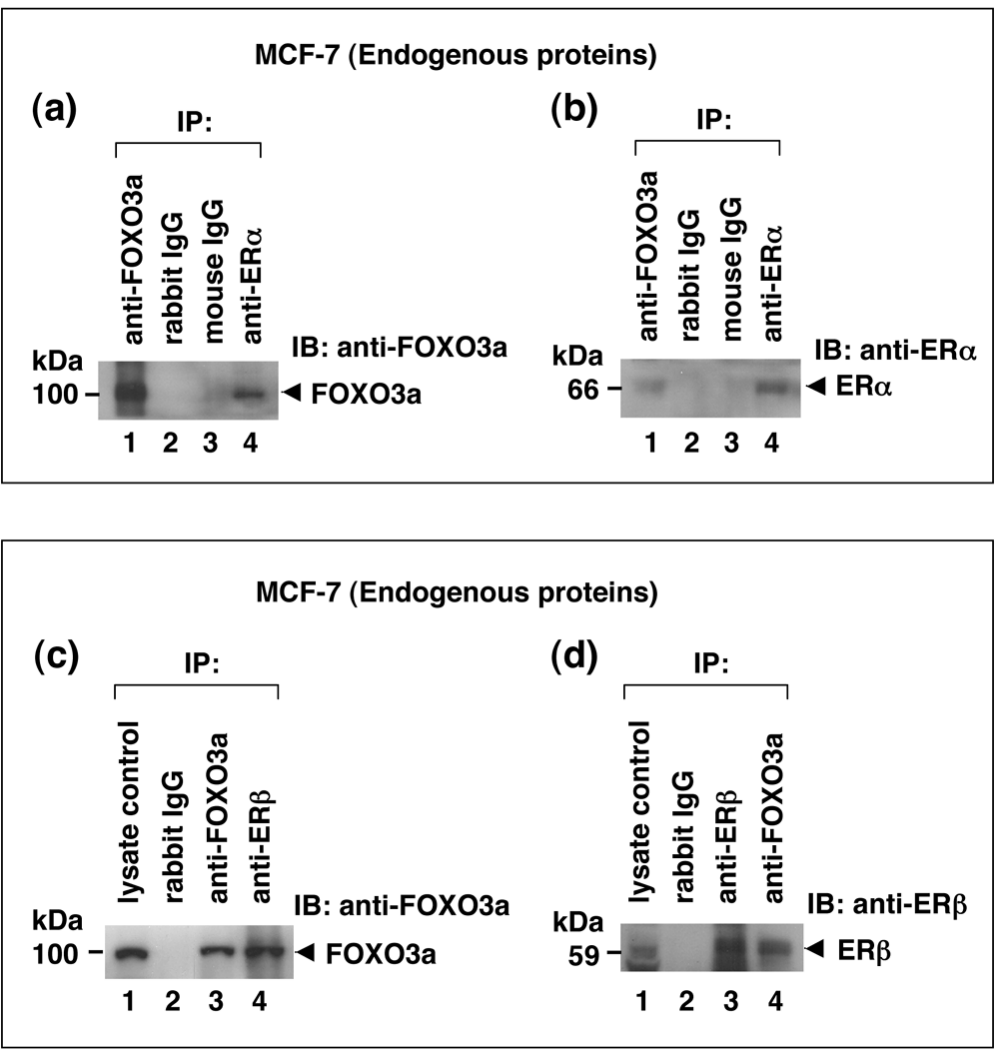
FOXO3a associates with endogenous ER-α and ER-β. **(a, b) **Forkhead box class O (FOXO)3a interacts with endogenous ER-α. Panel a: total lysates of MCF-7 cells with 17β-estradiol (E2) were subjected to immunoprecipitation (IP) with an anti-FOXO3a (positive control) or negative control IgGs (lanes 2 and 3) or anti-estrogen receptor (ER)-α (lane 4), followed by immunoblotting (IB) with an anti-FOXO3a antibody. Panel b: the same lysates of MCF-7 cells with E2 were subjected to reciprocal IP with an anti-FOXO3a antibody (lane 1) or a negative control IgG (lanes 2 and 3) or an anti-ER-α (positive control) followed by IB with an anti-ER-α. **(c, d) **FOXO3a interacts with endogenous ER-β. Panel c: total lysates of MCF-7 with E2 were subjected to IP/IB (IP: control IgG or anti-FOXO3a [positive control] or anti-ER-β (lane 4); IB: anti-FOXO3a). Panel d: total lysates of MCF-7 with E2 were subjected to a reciprocal IP/IB (IP: control IgG or anti-ER-β [positive control] or anti-FOXO3a [lane 4]; IB: anti-ER-β).

To investigate pathways that are modulated by FOXO3a, we performed a protein-protein interaction screen using an antibody array. We used cell lysates prepared from MCF-7 cells that were stably transfected with FOXO3a (MCF7-FO) to probe the antibody array and found that FOXO3a interacted with ER-α. We then confirmed the interaction between FOXO3a and ER using co-immunoprecipitation and immunoblotting assays. We found that FOXO3a physically interacted with ER-α and ER-β in the presence of E2 when we analyzed total lysates of 293T cells co-transfected with either HA-tagged FOXO3a plus Flag-ER-α or Flag-FOXO3a and HA-ER-β expression vectors with an anti-HA-specific or anti-Flag tag-specific antibody (Figure 1). We also used MCF-7 cells (an ER-positive cell line and requires the presence of E2 for growth in culture) and antibodies to show that endogenous FOXO3a associated with ER-α (Figure 2a,b) and ER-β (Figure 2c,d).

**Figure 3:**
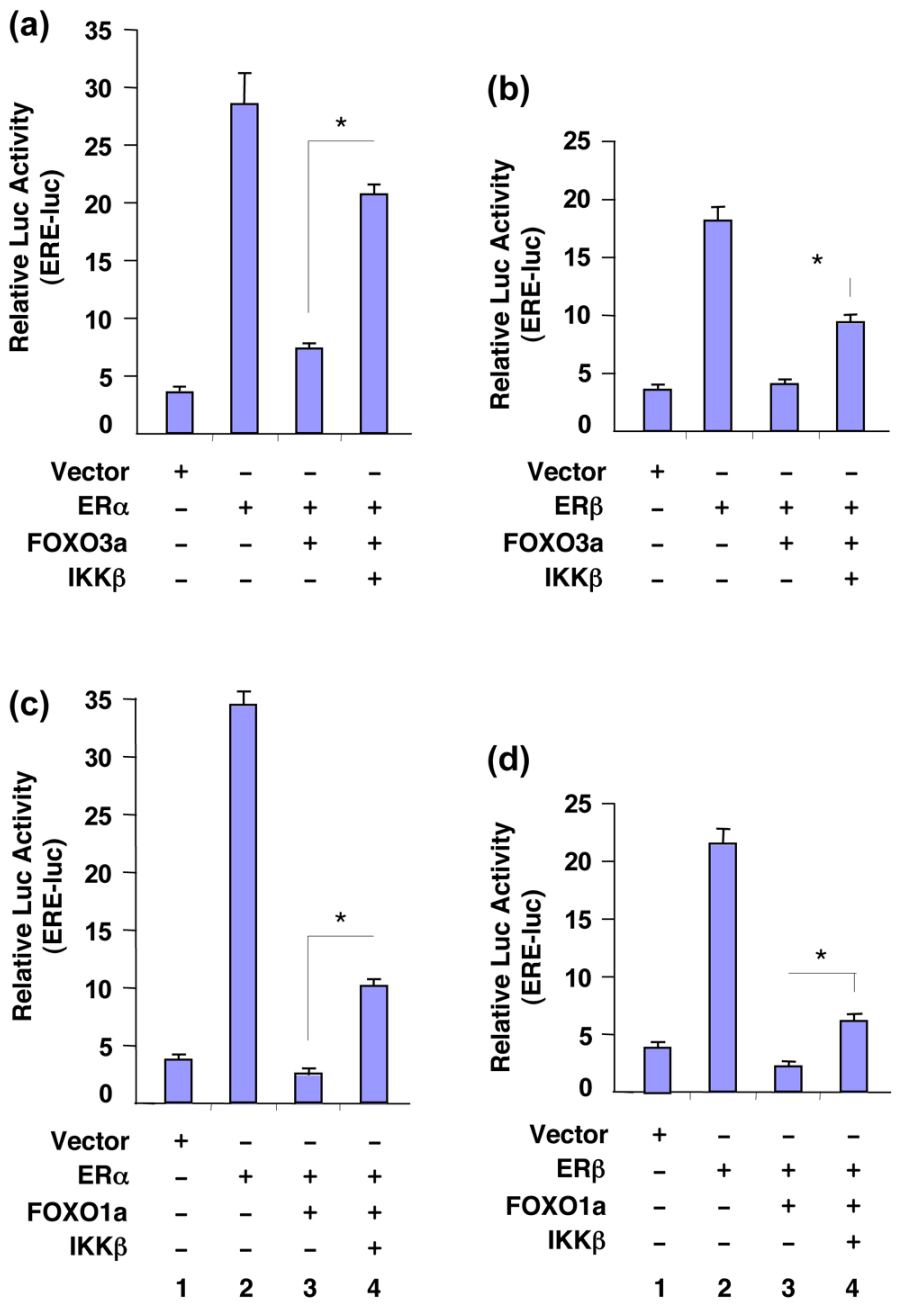
FOXO3a and FOXO1a inhibit the transactivation activities of ER-α and ER-β. **(a, b) **293T cells were co-transfected with estrogen receptor (ER)-responsive element (ERE)-luc (firefly luciferase [luc] reporter containing EREs), pRL-TK (renilla luc as a transfection control for normalization), ER-α (panel a) or ER-β (panel b), and forkhead box class O (FOXO)3a plus IκB kinase (IKK)-β or an empty vector (control) as indicated. Total lysates of the transfected cells were prepared and subjected to luc assays. **(c, d) **Total lysates of 293T cells were co-transfected with ERE-luc, pRL-TK, ER-α (panel c) or ER-β (panel d), and FOXO1a plus IKK-β or an empty vector as indicated and subjected to luc assays. All cells were cultured in the presence of 17β-estradiol (E2). The relative reporter luc activity was normalized with pRL-TK. Data are expressed means and standard deviations from three repeated experiments, which were performed independently. *P < 0.05 between FOXO (FOXO3a or FOXO1a) minus IKK-β (lane 3) versus FOXO plus IKK-β (lane 4).

FOXO3a interacts with and inhibits the transactivation activity of ER-α and ER-β We then tested the effect of FOXO3a on the ER-dependent transcriptional activation of a promoter containing EREs driving a luc gene. Co-transfection of FOXO3a together with ER-α or ER-β into 293T cells resulted in strong inhibition of ER-α or ER-β activity. Because IKK-β is known to inhibit FOXO3a activity potently [21], we cotransfected FOXO3a and ER-α or ER-β together with IKK-β and found that IKK-β restored much of the activity of ER-α or ER-β on the ERE-luc reporter (Figure 3a,b). These findings support the conclusion that repression of ER-α or ER-β activity on the ERE-luc reporter takes place specifically through FOXO3a. We also examined the effect of a different FOXO factor, namely FOXO1a, on ER-α and ER-β activity using the same co-transfection approach and found similar results (Figure 3c,d). These findings suggest that FOXO1a can also specifically inhibit the activity of ER-α or ER-β.

### FOXO3a suppresses ER-mediated signaling and upregulates CDK inhibitors in MCF-7

To investigate the mechanism by which FOXO3a might regulate ER-mediated signaling in breast cancer cells, we used DNA microarray and compared gene expression profiles of FOXO3a expressing MCF7-FO33 with the control MCF7-C5 (transfected with an empty vector) cells. The results indicated the downregulation of expression of certain ER-regulated genes, including those for PgR, cathepsin D, and complement C3, in the MCF7-FO33 cells, whereas the expression levels of ER-α and ER-β seemed unchanged. These findings suggest that FOXO3a suppresses ER transcriptional activity, perhaps by acting on ER-α or ER-β protein.

**Figure 4:**
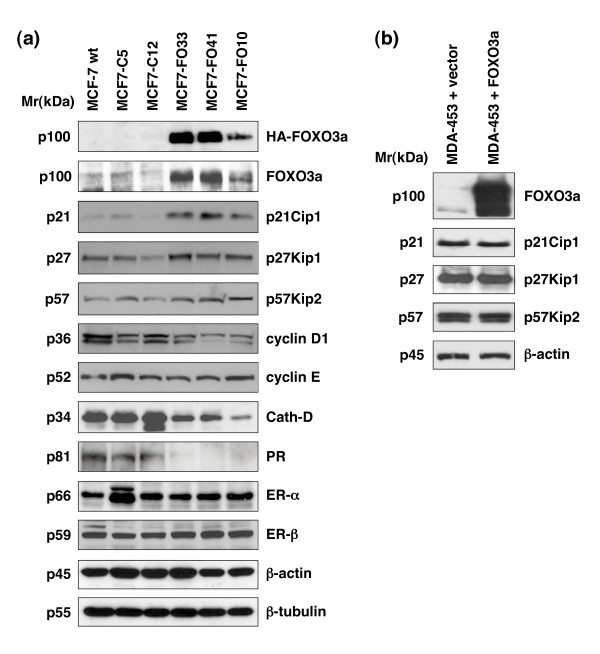
FOXO3a regulates expression of ER target genes and CDK inhibitors, and induces apoptosis in MCF-7. **(a) **Ectopic expression of Forkhead box class O (FOXO)3a reduces the expression of some estrogen receptor (ER)-regulated genes and enhances the expression of cyclin-dependent kinase (CDK) inhibitors in MCF7-FO cells in the presence of 17β-estradiol (E2). Immunoblotting (IB) analyses for HA-FOXO3a, endogenous FOXO3a, p27Kip1, p21Cip1, p57Kip2, cyclin D_1_, cyclin E, cathepsin D, progesterone receptor (PgR), ER-α, and ER-β protein expression in MCF7-FO33 and MCF7-FO41 cells (constitutively expressing FOXO3a) and in control (MCF-7 and MCF7-C5) cells were performed with specific antibodies, as indicated. Equal loading was confirmed by the same IB analysis with antibodies against β-actin or β-tubulin. **(b) **MDA-MB-453 (MDA-453, ER-negative) cells were transfected with either FOXO3a or an empty pCDNA3.1 vector (control), as indicated. Total lysates of the transfected cells were prepared and subjected to SDS-PAGE followed by IB analysis with the indicated antibodies.

ER-mediated signaling is generally considered critical for the survival and proliferation of estrogen-dependent breast cancer cells. To examine the long-term effects of FOXO3a on ER function in estrogen-dependent breast cancer cells and to elucidate the molecular mechanisms underlying those effects, we established three MCF7-FO stable cell lines over-expressing FOXO3a (MCF7-FO10, MCF7-FO33, and MCF7-FO41) and three control cell lines (MCF7-C4, MCF7-C5, and MCF7-C12). We then used immunoblotting to investigate whether constitutive expression of FOXO3a in the MCF7-FO cells affected FOXO3a transcriptional targets and ER-mediated signaling. Because p27Kip1 is known to be upregulated by FOXO3a through a transcriptional control mechanism [18,20] and CDK inhibitors are known to play important roles in controlling cell cycle and growth, we examined whether three CDK inhibitors (p27Kip1, p21Cip1, and p57Kip2) were regulated by FOXO3a in MCF7-FO. Interestingly, FOXO3a induced the expression of three CDK inhibitors in three MCF-FO cell lines compared with the control (Figure 4). These findings suggest a mechanism by which FOXO3a may induce breast cancer cell growth arrest through upregulation of all three key CDK inhibitors, which may be the direct transcriptional targets of FOXO3a.

Although FOXO3a significantly inhibited the expression of certain ER-regulated genes that are involved in cell growth or survival (for example, those encoding cathepsin-D, PgR, and cyclin D_1_) in the MCF7-FO, it did not alter the expression (Figure 4) or the cellular localization (data not shown) of ER-α and ER-β in those cells. In addition, we found that the expression of cyclin D_1 _was reduced, but cyclin E (Figure 4) and CDK2 and CDK4 (data not shown) were not significantly different between MCF7-FO and MCF7-C cells.

Because FOXO3a can promote apoptosis by upregulating FasL expression [18-20,28,31], we also examined whether FasL or Fas receptor (FasR) was induced by over-expression of FOXO3a in MCF7-FO. However, no differences in expression of FasL or FasR were observed (data not shown), suggesting that the FasR/FasL signaling pathway may not be involved in FOXO-mediated apoptosis in MCF7-FO cells.

### FOXO3a represses proliferation of MCF-7 cells *in vitro*

**Figure 5:**
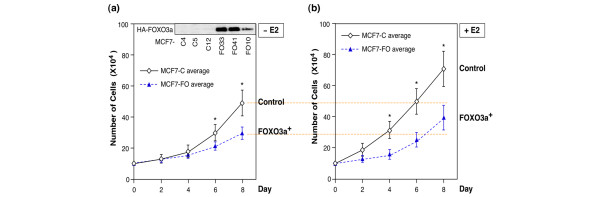
Ectopic expression of FOXO3a in estrogen-dependent breast cancer cells suppresses cell proliferation in cell culture. **(a) **Growth of forkhead box class O (FOXO)3a over-expressing MCF7-FO (MCF7-FO10, MCF7-FO33, and MCF7-FO41) cells and control (MCF7-C4, MCF7-C5, and MCF7-C12) cells in the absence of 17β-estradiol (E2) was determined by counting trypan-blue stained cells with a hemocytometer. Growth curves are the means of the three MCF7-FO cell lines (MCF7-FO10, MCF7-FO33, and MCF7-FO41) and the three control cell lines (MCF7-C4, MCF7-C5, and MCF7-C12); error bars indicate standard deviation from three experiments. **(b) **Growth of the same sets of cells in the presence of E2 (1 nmol/l). Growth curves are the means of the three MCF7-FO cell lines (designated MCF7-FO average) and the three control cell lines (designated MCF7-C average); error bars indicate standard deviaton from three experiments. *P < 0.05 between control MCF7-C group versus MCF7-FO group.

The MCF-7 cell line expresses ER-α and ER-β, and its proliferation requires estrogen stimulation. Hence, we sought to examine the effect of FOXO3a on the proliferation of MCF-7 cells in the absence or presence of E2. Direct cell counts showed that over-expression of FOXO3a significantly suppressed the proliferation of MCF7-FO cells in culture, both with and without E2. In the absence of E2 the average growth rate of MCF7-FO10, MCF7-FO33, and MCF7-FO41 cells was 38% (± 5%) less than that of the control MCF7-C4, MCF7-C5, and MCF7-C12 cells (*P *= 0.002; Figure 5a). In the presence of E2 the average growth rate of these three MCF7-FO cell lines was reduced by 47% (± 11%; *P *= 0.008; Figure 5b). These findings indicate that proliferation of human estrogen-dependent breast cancer cells is suppressed by the constitutive expression of FOXO3a.

### FOXO3a suppresses E2-dependent tumor growth of MCF-7 *in vivo*

**Figure 6:**
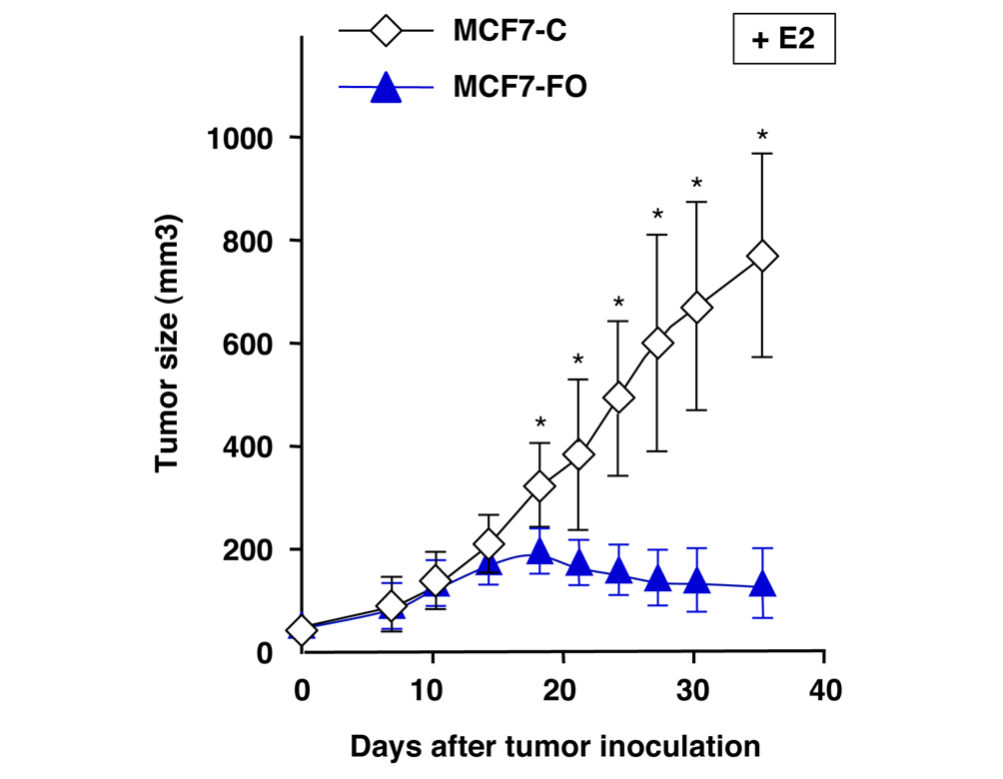
Ectopic expression of FOXO3a in estrogen-dependent breast cancer cells suppresses breast tumor development *in vivo*. Forkhead box class O (FOXO)3a suppresses estrogen receptor (ER)-positive breast tumor development in a mouse model of breast cancer. The MCF7-FO pooled cell lines and the control MCF7-C pooled cell lines were injected (2 × 10^6 ^cells/mouse) into the mammary fat pads of female athymic mice given supplementary 17β-estradiol (E2), as described in Materials and methods. Growth curves of tumor size are the means of the MCF7-FO pooled cell lines (designated MCF7-FO) and the control MCF7-C pooled cell lines (designated MCF7-C); error bars indicate standard deviation from three experiments. *P < 0.05 between control MCF7-C group versus MCF7-FO group.

The antiproliferative effect of FOXO3a on MCF-7 *in vitro *raised the possibility that constitutive expression of FOXO3a might suppress tumor growth *in vivo*. Because MCF-7 is an E2-dependent breast tumor cell line, no breast tumors were detected when mice were not given E2 (data not shown). When the control MCF7-C pooled cell lines were injected into the mammary fat pads of female athymic mice given supplemental E2, breast tumors appeared in approximately 2 weeks (Figure 6). Injection of the MCF7-FO pooled cell lines produced small tumors within about 2 weeks, but those tumors did not grow thereafter (Figure 6). Overall, after 5 weeks the average tumor growth of MCF7-FO pooled cells was 87% (± 21%) less than that of the control cell lines (*P *< 0.001) in the presence of E2. These results indicate that constitutive expression of FOXO3a in estrogen-dependent breast cancer cells significantly suppresses E2-dependent tumor growth *in vivo *in this orthotopic mouse model of breast cancer.

### FOXO3a reduces the expression of certain ER-regulated genes and increases the expression of CDK inhibitors in MCF7-FO breast tumors

**Figure 7:**
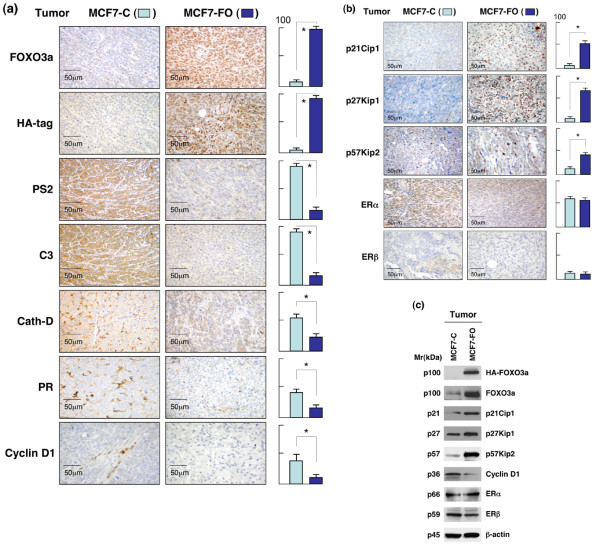
FOXO3a decreases expression of ER-regulated genes and increases CDK inhibitors in MCF7-FO breast tumors. **(a) **At 35 days after tumor cell implantation, breast tumors derived from female athymic mice bearing MCF7-FO or MCF7-C (control) tumors were resected, fixed, sectioned, and placed on slides. Five independent tumors (each from a different mouse) were tested in each mouse group. Tumor specimens were subjected to immunohistochemical (IHC) staining with antibodies specific to forkhead box class O (FOXO)3a, hemagglutinin (HA)-tag, pS2, complement C3, cathepsin (Cath)-D, progesterone receptor (PgR), and cyclin D_1_. Slides were examined at 40× magnification with a microscope. Numbers of positively staining cells in four random fields were counted in each tumor section, and representative fields are shown. Scale bars indicate 50 μm. *P < 0.01 between control MCF7-C group versus MCF7-FO group. **(b) **At 35 days after tumor cell implantation, breast tumors derived from female athymic mice bearing MCF7-FO or MCF7-C (control) tumors were resected, fixed, sectioned, and placed on slides. Five independent tumors (each from a different mouse) were tested in each mouse group. Tumor specimens were subjected to IHC staining with antibodies to p21Cip1, p27Kip1, p57Kip2, estrogen receptor (ER)-α, and ER-β. Numbers of positively staining cells in four random fields were counted in each tumor section, and representative fields are shown. Scale bars indicate 50 μm. *P < 0.02 between control MCF7-C group versus MCF7-FO group. **(c) **Confirmation that FOXO3a enhances expression of cyclin-dependent kinase (CDK) inhibitors and reduces expression of cyclin D_1 _in tumors in immunoblotting (IB) analysis. Whole lysates of MCF7-FO or MCF7-C breast tumor specimens were subjected to IB analysis with antibodies to p21Cip1, p27Kip1, p57Kip2, cyclin D_1_, ER-α, ER-β, HA-tag and FOXO3a (positive controls), and β-actin (loading control).

We then examined whether FOXO3a could regulate ER-mediated signaling molecules and CDK inhibitors in MCF7-FO *in vivo*. Immunohistochemical staining of the xenograft tumor sections showed that FOXO3a had repressed the expression of the ER target genes pS2, complement C3, cathepsin-D, and PgR (Figure 7a), and upregulated the expression of p21Cip1, p27Kip1, and p57Kip2 in tumors derived from MCF7-FO cells relative to tumors derived from control MCF7-C cells (Figure 7b). In accordance with our previous immunoblotting findings (Figure 4a), the expression levels of ER-α and ER-β were largely unchanged between MCF7-C and MCF7-FO tumor specimens. We also used immunoblotting of total cell lysates to confirm the upregulation of p21Cip1, p27Kip1 and p57Kip2, and the downregulation of cyclin D_1 _in MCF7-FO tumors relative to MCF7-C tumors (Figure 7c). Similarly, the expression of ER-α and ER-β proteins in total cell lysate was no different in the MCF7-FO and MCF7-C tumor samples, supporting the notion that FOXO3a may inhibit ER-mediated signaling pathways through a nontranscriptional mechanism *in vivo*. Collectively, these findings suggest that FOXO3a upregulates the expression of p21Cip1, p27Kip1 and p57Kip2, and inhibits ER-mediated signaling in MCF-7; these effects may lead to growth suppression in MCF7-FO tumor cells *in vivo*. Taken together, these findings suggest that FOXO3a may suppress tumor growth through inhibition of ER function or cell growth control in estrogen-dependent breast cancer *in vivo*.

### ER-α and ER-β bind to the unique domains of FOXO3a and FOXO3a downregulates FOXM1

**Figure 8:**
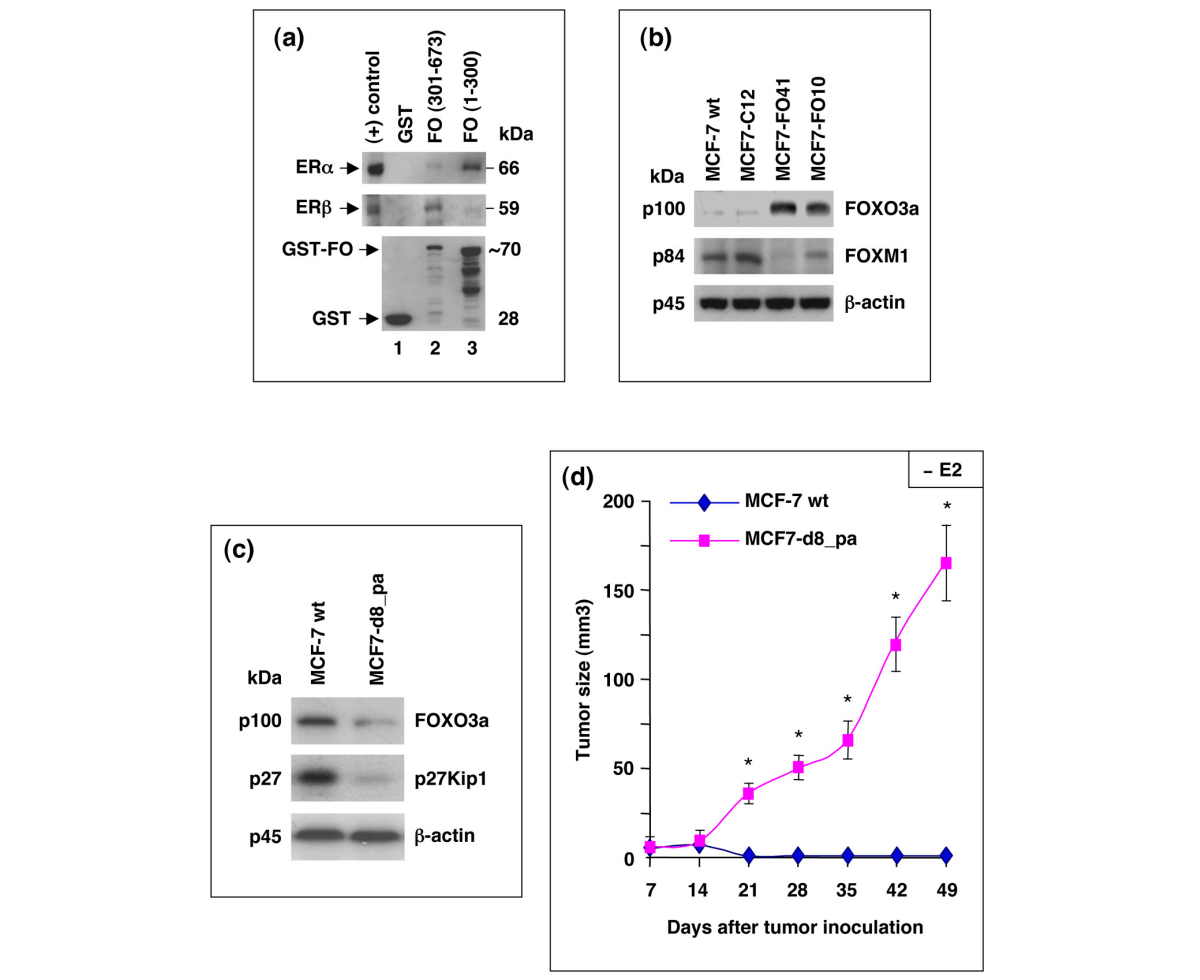
ER-α and ER-β bind to the amino-terminal and carboxyl-terminal domains of FOXO3a, respectively. **(a) **Glutathione-S-transferase (GST) – pull down *in vitro *assays. Whole cell lysates from 293T cells were incubated with the GST-forkhead box class O (FOXO)3a (GST-FO [amino acids 1 to 300] and GST-FO [amino acids 301 to 673]) fusion proteins as indicated and GST alone (negative control), and analyzed by SDS-PAGE and immunoblotting with an antibody (Ab) against estrogen receptor (ER)-α or ER-β (upper panels) and an anti-GST Ab (lower panel) as protein controls. **(b) **FOXO3a downregulates FOXM1. Immunoblotting (IB) analyses for endogenous FOXO3a, forkhead box M1 (FOXM1), and β-actin (loading control) protein expression in MCF7-FO10 and MCF7-FO41 cells (constitutively expressing FOXO3a) and in control (MCF-7 wt and MCF7-C12) cells were performed with specific antibodies antibodies as indicated. **(c) **MCF7-d8_pa cells (pooled clones of MCF-7 FOXO3a-knockdown derivatives) were established with retroviruses expressing small hairpin RNA against human FOXO3a. The expression levels of FOXO3a and p27Kip1 in MCF-7 wild-type (wt) and MCF7-d8_pa cells were determined by IB with specific Abs against FOXO3a or p27Kip1 or β-actin (loading control). **(d) **Silencing endogenous FOXO3a in MCF-7 cells promoted tumorigenesis *in vivo*. The tumor growth rates of control group MCF-7 wt and knockdown group MCF7-d8-pa were determined after injection of cells (2 × 10^6^cells/mouse) as indicated into the mammary fat pads of female athymic mice not given supplemental 17β-estradiol (E2; indicated as – E2). Data are expressed as means and standard deviations from two experiments with five mice in each group. *P < 0.01 between MCF7-d8-pa group versus control MCF-7 wt group.

To elucidate the candidate sites on FOXO3a by which FOXO3a interacts with ER-α and ER-β to regulate their functions, we examined the binding between FOXO3a and ER-α or ER-β by using standard Glutathione-S-transferase (GST) pull-down assays. The results showed that the amino-terminal domain (amino acids 1 to 300) of FOXO3a binds to ER-α whereas the carboxyl-terminal domain (amino acids 301 to 673) of FOXO3a binds to ER-β primarily (Figure 8a).

Because it was recently shown that FOXO3a can interact with forkhead box m1 (FOXM1) on ER-α promoter and regulate ER-α expression [36,37], we sought to determine whether over-expression of FOXO3a could affect FOXM1 expression. Our results showed that the level of FOXM1 protein was downregulated in FOXO3a-over-expressing MCF-7 cell lines (MCF7-FO41 and MCF7-FO10) as compared with that in the control MCF7-C cells (MCF-7 wild-type and MCF7-C12; Figure 8b). These results are consistent with those previous findings [36,37] suggesting that FOXO3a may repress ER-α activity through an alternative mechanism by which FOXO3a downregulates FOXM1 expression.

### Silencing endogenous FOXO3a in MCF-7 cells promoted tumorigenesis *in vivo*

To further confirm the regulatory role played by endogenous FOXO3a in suppressing tumor development and growth in the orthotopic breast tumor mouse model, we generated MCF7-d8_pa cells (pooled clones of MCF-7 FOXO3a knockdown derivatives) by using retroviruses expressing short hairpin RNA against human FOXO3a (Figure 8c). After injection of MCF7-d8_pa or MCF-7 wild-type (control) cells (2 × 10^6 ^cells/mouse) into the mammary fat pads of female athymic mice not given supplemental E2, the tumor growth rates of these cells were determined. Our results showed that silencing of FOXO3a indeed promoted tumor growth or tumorigenesis of MCF7-d8_pa cells in female athymic mice not given supplemental E2, whereas wild-type MCF-7 cells could not grow tumors in athymic mice in the absence of E2 (Figure 8d). Collectively, these results unambiguously confirm the tumor suppression role of FOXO3a in MCF7 cells and suggest a role for FOXO3a in preventing hormone-independent growth of MCF7 cells *in vivo*.

## Discussion

Here, we investigated the functional role of FOXO3a in estrogen-dependent breast cancer and showed that constitutive expression of FOXO3a in MCF-7 suppressed proliferation *in vitro *and estrogen-dependent breast tumor development *in vivo *in an orthotopic breast cancer model. Strikingly, silencing endogenous FOXO3a converted nontumorigenic, estrogen-dependent MCF-7 cells into tumorigenic estrogen-independent cells, supporting the concept that FOXO3a plays a critical tumor suppression role in estrogen-dependent breast cancer. These results are consistent with recent findings that cancers develop in mice that lack *FOXO *genes [38,39], indicating that the FOXO molecules are *bona fide *tumor suppressors in mammals. Reasoning that these findings might be extended to the identification of agents (such as small molecules) that can activate FOXO3a for development as a new tumor suppressive therapeutic modality in breast cancer, we studied the mechanisms by which FOXO3a suppressed the proliferation and tumorigenicity of MCF-7.

p27Kip1 is known to be a transcriptional target of FOXO factors [18-20], and Smad-FOXO complexes can induce p21Cip1 expression [40]. Interestingly, we found that FOXO3a upregulated the expression of all three CDK inhibitors tested (p21Cip1, p27Kip1, and p57Kip2; Figure 4a). This finding is novel and important because FOXO3a has been shown to regulate entry into and exit from mitosis [26], which is critical in regulating cell proliferation or apoptosis in normal and cancerous cells. These findings also confirm our observations that FOXO3a inhibited the growth of estrogen-dependent breast cancer cells both in culture and in an orthotopic mouse model of breast cancer, suggesting that FOXO3a is involved in suppressing tumor growth or tumorigenesis and ER-mediated signaling in ER-positive breast tumors *in vivo*.

Downregulation of cyclin D was previously reported to be involved in FOXO-induced cell cycle inhibition in some cancer cell types such as colon carcinoma cell lines [27]. Because it has been shown that D-type cyclins are regulated by estrogen and ER, and play important roles in controlling cell growth [41], we compared the expression of cyclins D_1_, D_2_, and D_3 _between MCF7-FO cells and the control MCF7-C cells. In agreement with their findings, we found that the expression of cyclin D_1 _was reduced in MCF7-FO cells as compared with that in control MCF7-C cells (Figure 4a). However, we could not detect any significant difference in the expression of cyclins D_2 _and D_3 _between MCF7-FO cells and the control MCF7-C cells (data not shown), suggesting that downregulation of cyclins D_2 _and D_3 _may not be involved in FOXO3a-induced growth arrest in MCF-7 breast cancer cells. In addition, we found that the expression of cyclin E is not significantly altered in these MCF7-FO cells either (Figure 4a). These results suggest that cell growth arrest induced by FOXO3a may be primarily through upregulation of key CDK inhibitors instead of downregulation of cyclins in MCF7-FO cells.

It was previously shown that ER-α interacts with FOXO3a (FKHRL1) in a ligand-dependent manner, and the ligand-dependent ER-α-FKHR interaction can be inhibited by tamoxifen [42]. However, those data suggest that FKHR-TM activates ER-α-mediated transcriptional activity. In contrast, another report [43] demonstrated that FKHR interacts with ER-α and represses ER-α-mediated transactivation. Both studies [42,43] were mainly based on transient co-transfection reporter experiments. The differences between the two studies can be attributed to the different vectors or approaches used. Because the regulation of ER-α plays a pivotal role in breast cancer and these two reports appear to be contradictory in their findings, further investigation of the role of FOXO in regulating ER function *in vivo *is certainly justified and necessary.

Using orthotopic breast cancer xenograft mouse models, for the first time we clearly showed that over-expression of FOXO3a in MCF-7 cells suppressed their ER-dependent tumorigenesis and growth *in vivo*. Our results support that FOXO3a represses the transcriptional activity of ER-α, as was described by Zhao and coworkers [43]. Both studies show that FOXO represses the transcriptional activity of ER-α in an interaction that is dependent on E2 (an agonist). In addition, we show that FOXO3a also suppresses the transcriptional activity of ER-β. This is important in breast cancer cells because, under physiological conditions, ER appears to be positively regulated by estrogen in breast cancer cells. However, when FOXO3a is active, FOXO3a can antagonize the estrogen-dependent functions of ER-α and ER-β, resulting in suppression of ER-mediated tumor growth and development.

We further investigated the role played by FOXO3a in inhibiting ER-mediated signaling and growth of the estrogen-dependent breast cancer cells. Our finding that silencing FOXO3a in MCF-7 promoted tumorigenesis and tumor growth in an E2-independent manner *in vivo *(Figure 8d) further supports the notion that FOXO3a plays a critical role in repressing the ER-mediated survival pathway *in vivo*. One plausible mechanism of this effect is that ER becomes constitutively active in the absence of estrogen (such as the development of hormone-refractory disease) when MCF-7 cells are lacking FOXO3a, an ER co-repressor. The second possible mechanism of this effect is that silencing of FOXO3a might induce certain receptor tyrosine kinases, which in turn activate ER activity in MCF-7 cells in an estrogen-independent manner. The third possible mechanism of this effect could be that other factors (such as cytokines or chemokines) might be upregulated in MCF-7 cells that lack FOXO3a through autocrine regulation and contribute to this hormone-independent growth effect. However, we have no mechanistic data to prove the possible mechanism of this effect at present. Further elucidation of the molecular mechanism that underlies this effect will lead to better understanding of the role of FOXO3a in preventing hormone-independent growth of MCF7 cells *in vivo*.

Notably, we also demonstrated a direct physical interaction between FOXO3a and ER-α or ER-β and suppression of ER-α and ER-β transactivation activities by FOXO3a *in vitro*. Transient over-expression of FOXO3a has been shown to lead to increases in ER-α expression and ER-α promoter activity in a reporter assay in ER-α-positive NF639 cells [44]. However, we found that the expression of ER-α and ER-β proteins was not significantly affected by the ectopic expression of FOXO3a in all three variants (MCF7-FO) of ER-α-positive and ER-β-positive MCF-7 cells. One possible explanation for this difference is that FOXO3a can function on the one hand as a co-repressor, one that is associated with ER-α and ER-β proteins and inhibits their transactivating activities; and on the other hand as a transcription factor that binds the promoter of ER-α and induces transcription of ER-α as a feedback mechanism in some situations. Alternatively, the observed difference could be due to differences in the expression methods or the cell lines used.

Because many estrogen-dependent breast cancer cells express both ER-α and ER-β, one plausible meaning of this observation is that FOXO may interact with ER-α and ER-β simultaneously at different FOXO domains, resulting in inhibition of both ER-α and ER-β function in breast cancer cells and suppression of ER-mediated tumor growth and development. In general, ER-α and ER-β consist of six functional domains. The structurally distinct amino terminal A/B domains (17% amino acid identity) contain a ligand-independent transactivation function (AF1). The near identical central C region is the DNA-binding domain. The flexible hinge, or D, domain contains a nuclear localization signal and links the C domain to the multifunctional carboxyl-terminal (E/F) domain, which exhibits 56% amino-acid homology between ER-α and ER-β. E/F is involved in ligand binding, dimerization, and ligand-dependent transactivation functions (AF2). Thus, the second possible significance of this phenomenon is that ER-α and ER-β may use distinct functional domains to interact with FOXO at different domains so that ER-α and ER-β may not compete for the binding site of FOXO. Moreover, it has been shown that ER-α and ERβ can form heterodimers on DNA, and they interacted with ERE and E2 in a manner similar to that observed with the ER homodimers [45]. The third possible implication of this finding is that ER-α and ER-β heterodimers may interact with FOXO at different domains to form a large complex that could not bind to ERE on DNA, and thereby ER-α/ER-β are not functional.

Finally, our findings provide a mechanistic basis for FOXO3a-mediated tumor suppression in ER-positive breast cancer cells. FOXO3a inhibited ER-mediated signaling through a nongenomic mechanism and upregulated the expression of three CDK inhibitors that could result in suppression of tumor growth and tumorigenesis in estrogen-dependent breast cancer cells *in vivo*. Although our results were generated using MCF-7 variants and mouse models, the *in vivo *data in particular may have important clinical implications for treating or preventing the development of resistance to endocrine therapy. For instance, the development of resistance to antiestrogen therapy remains a clinically important problem. However, a major limitation in alternative approaches to treating or preventing antiestrogen resistance is that we lack knowledge of the precise signaling mechanisms that underlie the regulation of ER function and development of ER-unresponsiveness in ER-positive breast cancer cells *in vivo*. Further investigation of FOXO factors in inhibition of ER function and signaling may contribute new insights into this problem. Understanding the molecular basis of FOXO3a-induced suppression of cell growth and tumor development could also provide opportunities to develop innovative anticancer therapeutic modalities, such as small molecules that can activate FOXO3a, thereby potentially suppressing the growth of breast tumors and possibly preventing recurrence after therapy.

## Conclusion

We suggest that FOXO3a plays a critical role in suppressing estrogen-dependent breast cancer cell growth and tumorigenesis *in vivo*. Our data further support that agents such as small molecules that activate FOXO3a may be novel therapeutics for inhibition and prevention of tumor proliferation and development in breast cancer.

## Abbreviations

CDK = cyclin-dependent kinase; E2 = 17β-estradiol; ER = estrogen receptor; ERE = ER-responsive element; FasL = Fas ligand; FasR = Fas receptor; FOXO = forkhead box class O; FOXM1 = forkhead box M1; HA = hemagglutinin; IKK = IκB kinase; luc = luciferase reporter; PBS = phosphate-buffered saline; PgR = progesterone receptor.

## Competing interests

The authors declare that they have no competing interests.

## Authors' contributions

YZ conducted all of the breast tumorigenesis and tumor growth studies in female athymic nude mice. W-BT performed the co-immunoprecipitation, immunoblotting, and antibody array experiments. C-JC conducted immunohistochemical staining of MCF-7 derived breast tumors. P-CL performed the ER-mediated luciferase reporter assays. CH and YMC conducted part of immunoblotting analyses. S-HL provided critical suggestions and helped to revise and edit the manuscript. MC-TH generated DNA constructs and cell lines, designed and coordinated all experimental approaches, and drafted and revised the entire manuscript.
